# Severe hyperbilirubinemia secondary to ECMO in a ARDS patient: a case report

**DOI:** 10.3389/fmed.2026.1709850

**Published:** 2026-02-10

**Authors:** Yamin Gao, Wei Wang, Yuchen Duan, Zhai Huang, Xiaogang Tang

**Affiliations:** 1 Department of Critical Care Medicine, The People’s Hospital of Guangxi Zhuang Autonomous Region, Nanning, China; 2 Guangxi Academy of Medical Sciences, Nanning, China

**Keywords:** acute respiratory distress syndrome, extracorporeal membrane oxygenation, hemolysis, hyperbilirubinemia, multidisciplinary management

## Abstract

**History:**

A 51-year-old male presented with a 9-day history of fever and cough, progressing to severe respiratory failure refractory to conventional management.

**Diagnostic methods:**

High-throughput sequencing confirmed influenza A virus. Chest CT revealed bilateral pneumonia and pulmonary edema. Due to a persistently low oxygenation index (PaO₂/FiO₂ 48.9 mmHg), veno-venous extracorporeal membrane oxygenation (VV-ECMO) was initiated.

**Treatment:**

The patient was managed with a lung-protective ventilation strategy, prone positioning, and appropriate antimicrobial therapy. During ECMO support, severe mixed hyperbilirubinemia with a significant hemolytic component developed, characterized by a peak total bilirubin of 887 μmol/L. Management of hyperbilirubinemia included liver-protective agents, dual plasma molecular adsorption system (DPMAS), and therapeutic plasma exchange. Immunohematological tests confirmed immune-mediated hemolysis (positive direct and indirect antiglobulin tests).

**Outcome:**

Following ECMO decannulation, bilirubin levels normalized. The patient was successfully weaned from mechanical ventilation, transferred to a general ward, and eventually discharged, achieving full social reintegration.

**Conclusion:**

This case highlights the diagnostic challenge of differentiating between mechanical and immune-mediated hemolysis in ECMO patients and demonstrates the successful application of a combined therapeutic approach for severe ECMO-associated hyperbilirubinemia.

## Introduction

Extracorporeal Membrane Oxygenation (ECMO) serves as a vital therapeutic modality for refractory cardiogenic shock and severe respiratory failure (1). Despite its life-saving potential, ECMO is associated with significant complications, including bleeding, thrombosis, renal failure, and hemolysis, which adversely affect patient outcomes (2, 3). Hyperbilirubinemia is a notable complication during ECMO support, often arising from hemolysis due to mechanical shear stress and hepatic dysfunction secondary to hypoperfusion or systemic inflammation. Elevated bilirubin can induce apoptosis, trigger inflammatory responses, and promote oxidative stress, contributing to multi-organ dysfunction (4). This article presents a case of severe acute respiratory distress syndrome (ARDS) caused by influenza A, complicated by hyperbilirubinemia during ECMO support. We aim to discuss the diagnosis and management of this case, with the goal of enhancing intensivists’ clinical management of ECMO patients and their ability to address hyperbilirubinemia, thereby potentially improving prognosis and survival rates.

## Clinical data

### General information

A 51-year-old male (height: 172 cm, weight: 70 kg) was admitted as an emergency on May 5, 2024, with a 9-day history of fever (up to 39 °C), cough, and dizziness. He denied cardiopulmonary or gastrointestinal symptoms. Initial treatment at a local clinic with intravenous fluids and antitussives was ineffective. Subsequent hospitalization revealed influenza A virus via high-throughput sequencing of blood and bronchoalveolar lavage fluid. Chest CT showed bilateral pneumonia, secondary pulmonary tuberculosis (calcified/proliferative foci in the right upper lobe), bilateral micronodules, and a small pericardial effusion. Treatment with high-flow oxygen therapy, antibiotics, and mucolytics failed to improve his oxygenation index (PaO₂/FiO₂ 48.9 mmHg). Endotracheal intubation with mechanical ventilation and prone positioning were initiated, yet oxygenation remained poor. Our ECMO team was consulted and performed VV-ECMO on-site at the local hospital before transferring him to our ICU.

#### Past medical history

The patient had a history of pulmonary tuberculosis (treated and cured in 2021), hypertension (controlled with nifedipine), and type 2 diabetes (controlled with metformin and traditional Chinese medicine).

#### Physical examination on ICU admission

The patient was sedated (Richmond Agitation-Sedation Scale [RASS] -3) and on mechanical ventilation. Vital signs: temperature 37.3 °C, heart rate 100 bpm, blood pressure 145/68 mmHg, SpO₂ 96%. Ventilator settings: SIMV mode, FiO₂ 40%, VT 300 mL, RR 10 breaths/min, PEEP 12 cmH₂O. VV-ECMO (Maquet Cardiohelp system, Getinge, Germany; HLS Set Advanced 7.0 oxygenator) parameters: speed 3,590 rpm, blood flow 4.49 L/min, sweep gas flow 3 L/min, FiO₂ 100%. Cannulation: 25 Fr femoral venous drainage cannula and 19 Fr jugular venous return cannula.

Examination revealed coarse breath sounds and prominent moist rales bilaterally. Heart sounds were distant; no murmurs were detected. The abdomen was soft and non-tender; bowel sounds were diminished. No peripheral edema was noted.

### Diagnostic investigations and laboratory trends

Initial laboratory tests upon ICU admission (ECMO Day 1) are summarized in Table 1. Key findings included leukocytosis (WBC 18.93 × 10^9^/L), elevated inflammatory markers (IL-6 99.34 pg./mL, PCT 10.08 ng/mL), and initial hyperbilirubinemia (Total Bilirubin 47 μmol/L). The activity of G6PD in red blood cells is 1,649 U/L. Arterial blood gas under ECMO (FiO₂ 100%) showed a P/F ratio of 180 mmHg.

**Table 1:** Key laboratory parameters during ECMO course.

Parameter (reference range)	Day 1 (initial rise)	Day 3 (peak)	Day 9 (recovery)
Total bilirubin (μmol/L)	226.6 ↑	887.0 ↑	40.6 ↑
Direct bilirubin (μmol/L)	119.8 ↑	674.4 ↑	24.9 ↑
Indirect bilirubin (μmol/L)	106.8 ↑	239.6 ↑	15.7
AST (U/L)	171 ↑	387 ↑	17
ALT (U/L)	8	25	5
LDH (U/L)	4,330 ↑	6,972 ↑	248
Hemoglobin (g/L)	180 ↑	146	98 ↓
Platelets (×10^9^/L)	179	442 ↑	375 ↑
Creatinine (μmol/L)	148 ↑	211 ↑	255 ↑
Lactate (mmol/L)	1.2	1.4	1.8
Direct antiglobulin test	Not done	Positive (IgG)	Not repeated
Indirect antiglobulin test	Not done	Positive (Autoab)	Not repeated
G6PD (U/L)	1,649	Not done	Not done

#### Imaging

**Figure 1:**
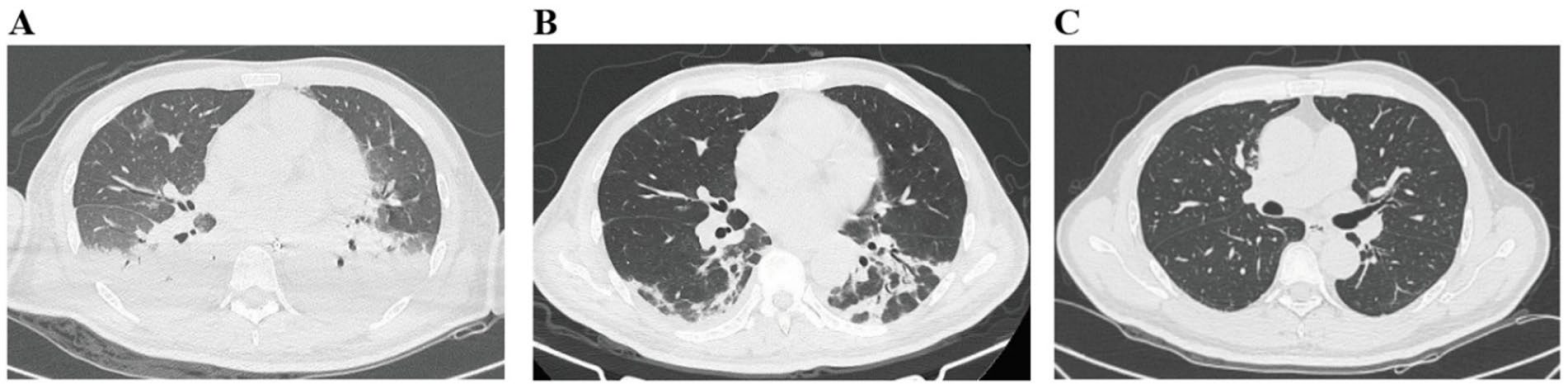
Improvement in lung imaging before and after standardized treatment, as shown on chest CT. Panel **A** shows a chest CT scan with patchy areas of haziness in both lungs. Panel **B** displays a chest CT scan with multifocal consolidations and ground-glass opacities, especially in the lower lung zones. Panel **C** depicts a chest CT scan of clear lungs with normal parenchymal markings and no significant opacities.

Chest X-ray and CT confirmed bilateral pulmonary infiltrates/edema. Abdominal ultrasonography suggested a hepatic hemangioma and biliary sludge (Figure 1).

### Diagnosis

#### Final diagnoses

1. Severe pneumonia (Influenza A virus).
2. Sepsis and Septic Shock.
3. Acute Respiratory Distress Syndrome (ARDS).
4. Respiratory Failure.
5. Mixed Hyperbilirubinemia with Significant Hemolytic Component.
6. Type 2 Diabetes Mellitus.

#### Diagnostic rationale for hyperbilirubinemia

The patient developed a severe, mixed hyperbilirubinemia (both conjugated and unconjugated fractions elevated) during ECMO support. Evidence supporting a significant hemolytic component included a dramatic rise in lactate dehydrogenase (LDH) to 6,972 U/L and a positive direct antiglobulin test (Coombs test), confirming immune-mediated hemolysis The initial mechanical stress from ECMO (e.g., circuit kinking during prone positioning leading to transiently high negative inlet pressures of −50 to −60 mmHg) was a potential inciting factor for red blood cell injury. The subsequent positive Coombs test suggested a possible secondary immune-mediated mechanism. Concurrent hepatic dysfunction, likely due to septic shock, systemic inflammation, and drug effects, impaired bilirubin conjugation and excretion, contributing to the mixed pattern. Bilirubin levels declined rapidly after ECMO decannulation.

### Treatment

Management included VV-ECMO support, lung-protective ventilation, daily prone positioning, sedation (Ciprofol, Remifentanil), vasopressors (Norepinephrine), antimicrobials (Imipenem-Cilastatin, Linezolid, Oseltamivir), and continuous renal replacement therapy (CRRT).

**Figure 2:**
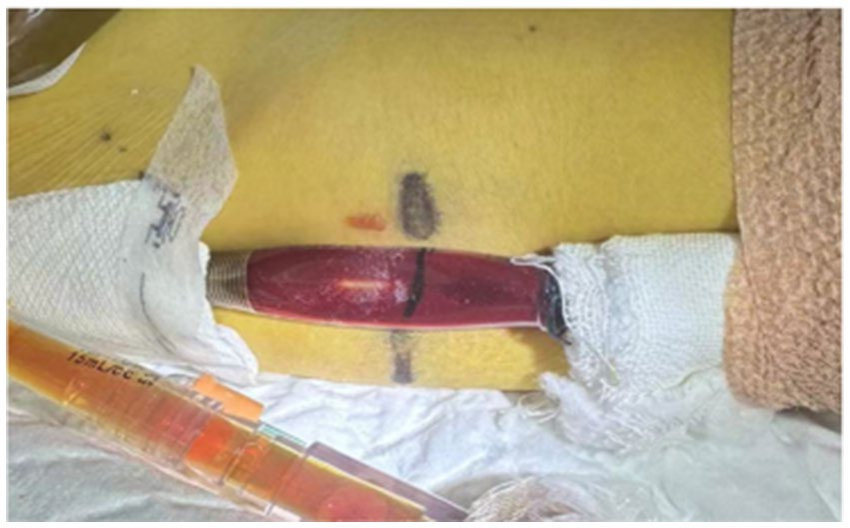
Deformation of the ECMO circuit observed during prone positioning.

On ECMO Day 2, bilirubin levels rose sharply, and scleral icterus was noted. Hepatoprotective agents (Ursodeoxycholic acid) were initiated. During a prone positioning session, circuit kinking occurred (Figure 2), causing transiently high negative inlet pressure and reduced flow, which was promptly corrected.

On Day 4 (Peak Bilirubin), DPMAS was initiated. Given the confirmation of immune-mediated hemolysis (positive Coombs tests) and persistent hyperbilirubinemia, therapeutic plasma exchange was performed on Day 5. After comprehensive evaluation, VV-ECMO support was successfully discontinued on Day 5.

**Figure 3:**
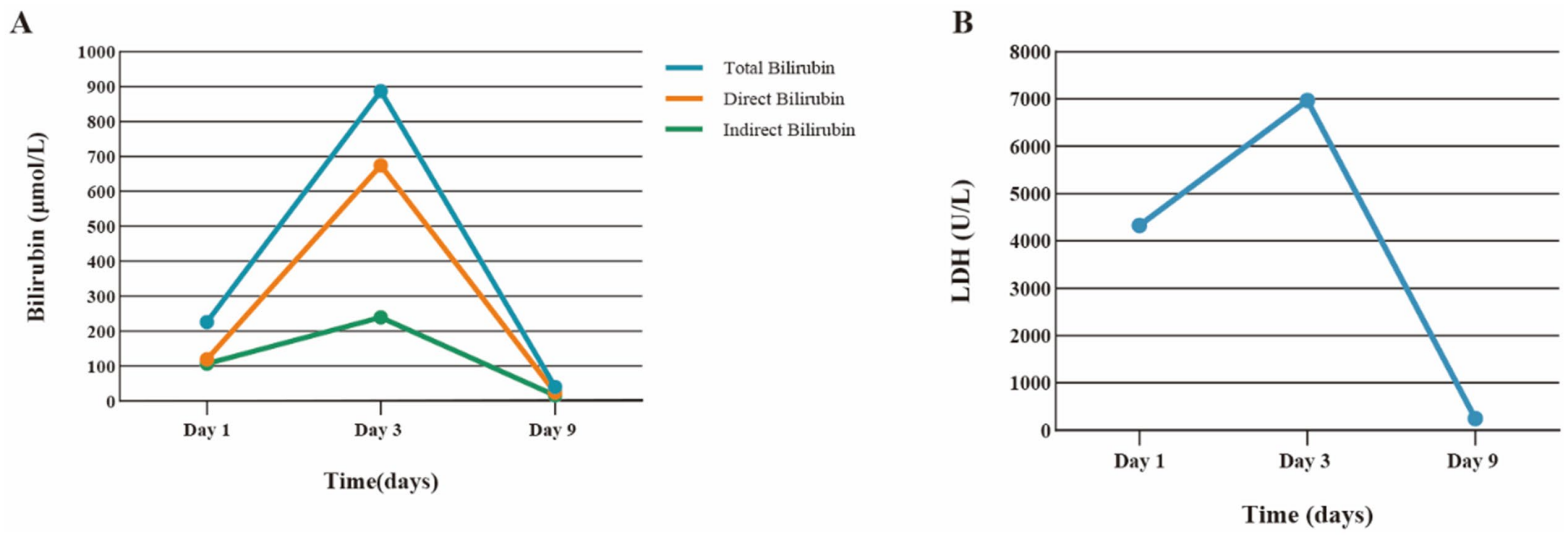
Plot timeline of total bilirubin, direct bilirubin, and LDH levels over time. Panel **A** displays a line graph comparing total, direct, and indirect bilirubin levels in micromoles per liter over days one, three, and nine, with all levels peaking on day three. Panel **B** shows a line graph of LDH levels in units per liter over the same days, also peaking on day three before declining sharply by day nine.

Bilirubin levels showed significant improvement thereafter (Figure 3). The patient was successfully extubated on Day 8. He was transferred to a general ward on Day 12 and discharged on Day 23. Follow-up on August 19 showed resolved abnormalities.

## Discussion

This case elucidates the diagnostic and therapeutic challenges of severe hyperbilirubinemia during ECMO support. The unique learning point is the progression from suspected mechanical hemolysis to confirmed immune-mediated hemolysis, necessitating a shift in management.

### Diagnostic challenge and etiology

Hyperbilirubinemia in ECMO patients is often multifactorial (5). Our patient presented a mixed picture. The initial dramatic rise in bilirubin and LDH, coupled with a circuit kinking event, pointed strongly towards mechanical hemolysis (6, 7). However, the positive Coombs test revealed an underlying immune-mediated component, a phenomenon that can be triggered by mechanical damage exposing neoantigens on red blood cells (8). This highlights that hemolysis in ECMO can be a spectrum, and immunohematological testing is crucial for a complete diagnosis. Concurrently, septic shock and systemic inflammation likely contributed to hepatic dysfunction, impairing the liver’s capacity to manage the bilirubin load from hemolysis, leading to the severe mixed hyperbilirubinemia (9, 10).

### Management strategy

Our approach was multifaceted. While circuit integrity was optimized, the confirmation of immune-mediated hemolysis justified the use of therapeutic plasma exchange, which effectively removes antibodies and inflammatory mediators (11). DPMAS provided adjunctive support by directly clearing bilirubin and toxins. CRRT aided in volume management and clearance of free hemoglobin. This combined strategy, tailored to the evolving diagnosis, was instrumental in managing this life-threatening complication (12).

## Conclusion

Severe hyperbilirubinemia during ECMO is a critical indicator of morbidity. This case underscores the importance of a systematic diagnostic approach to differentiate between mechanical and immune-mediated hemolysis. A high index of suspicion for immune-mediated mechanisms should be maintained, especially when hyperbilirubinemia is severe or disproportionate to the perceived mechanical insult. A multidisciplinary strategy, including close circuit monitoring, targeted extracorporeal liver support, and immunomodulation, can successfully manage this complex condition and improve outcomes.

## Data Availability

The raw data supporting the conclusions of this article will be made available by the authors, without undue reservation.
